# Osteoprotective effects of partially defatted house cricket (*Acheta domesticus*) powder in spontaneously hypertensive rats

**DOI:** 10.1371/journal.pone.0349511

**Published:** 2026-05-18

**Authors:** Kukiat Tudpor, Kasama Wongprachum, Tarinee Nilkamheang, Chaloemporn Namyota, Nitchara Toontom, Le Ke Nghiep, Khuanjit Chaimongkolnukul, Surachai Chantip, Nontawan Choovattanapakorn, Panan Suntornsaratoon, Kannikar Wongdee, Jarinthorn Teerapornpuntakit, Narattaphol Charoenphandhu, Sirithon Siriamornpun

**Affiliations:** 1 Faculty of Public Health, Mahasarakham University, Maha Sarakham, Thailand; 2 Public Health and Environmental Policy in Southeast Asia Research Cluster (PHEP-SEA) and Faculty of Public Health, Mahasarakham University, Maha Sarakham, Thailand; 3 Vinh Long Department of Health, Vinh Long, Vietnam; 4 National Laboratory Animal Center, Mahidol University, Nakhon Pathom, Thailand; 5 Center of Calcium and Bone Research (COCAB), Faculty of Science, Mahidol University, Bangkok, Thailand; 6 Department of Physiology, Faculty of Science, Mahidol University, Bangkok, Thailand; 7 Faculty of Allied Health Sciences, Burapha University, Chonburi, Thailand; 8 Physiology Division, Preclinical Science, Faculty of Medicine, Thammasat University, Pathum Thani, Thailand; 9 Institute of Molecular Biosciences, Mahidol University, Nakhon Pathom, Thailand; 10 The Academy of Science, The Royal Society of Thailand, Bangkok, Thailand; 11 Research Unit of Thai Food Innovation, Department of Food Technology and Nutrition, Mahasarakham University, Maha Sarakham, Thailand; University of Life Sciences in Lublin, POLAND

## Abstract

Clinical evidence suggests that essential hypertension is linked to oxidative stress and inflammatory infiltration, which can complicate osteoporosis. Edible house crickets (*Acheta domesticus*) are novel functional foods rich in proteins, fat, dietary fibers, and micronutrients. This research investigated the effects of a partially defatted house cricket powder (PDCP) on bone microarchitecture and strength in the spontaneously hypertensive rat (SHR) model. Fourteen female SHRs were divided into the experimental group receiving 300 mg/kg/day PDCP and the control group receiving the vehicle for 4 weeks. Blood pressure was determined by the CODA^®^ non-invasive blood pressure system. Femoral bone microarchitecture and strength were determined by micro-computed tomography (µCT) and three-point bending, respectively. Immune cells were counted using an automated machine. Results showed that in the control group, systolic blood pressure (SBP) at baseline (166.6 ± 3.7 mmHg) increased to 182.4 ± 4.7 mmHg (*P* = 0.016). In contrast, the PDCP-treated group showed no significant change from baseline (168.1 ± 3.9 mmHg) to post-intervention (176.9 ± 5.6 mmHg) (*P* = 0.156). PDCP had no effects on bone microarchitecture but improved bone strength. The post-intervention yield load (a proxy for strength) of the control group was 77.55 ± 1.88 N, compared to the PDCP-treated group of 83.58 ± 1.26 N (*P* < 0.009). Similarly, post-intervention yield displacement was 307.72 ± 29.78 in the control vs. 395.93 ± 40.98 µm in the PDCP-treated group. White blood cell counts in the PDCP-treated group (7.91 ± 0.27 × 10^3^/µL) were significantly higher than those in the control (6.91 ± 0.31 × 10^3^/µL). Specifically, lymphocyte counts in the PDCP-treated group (6.66 ± 0.22 × 10^3^/µL) were significantly higher than those in the control (5.75 ± 0.28 × 10^3^/µL). In conclusion, the 4-week PDCP had osteoprotective effects, presumably mediated by dietary proteins and fibers that modulate immune-vascular homeostasis, thereby potentially mitigating immune system-related hypertension and bone mechanical impairment.

## Introduction

Hypertension is a chronic condition that affects approximately 1.5 billion people worldwide [1]. According to recent study findings, hypertension has been demonstrated to be an inflammatory and oxidative stress-related disease characterized by an imbalanced number of immune cells, which results in various cytokine alterations, subsequently leading to endothelial dysfunction and organ damage [2]. On the one hand, regulatory T lymphocytes defend against hypertension by secreting anti-inflammatory cytokines, such as transforming growth factor-β (TGF-β) and interleukin-10 (IL-10) [3]. On the other hand, T and B lymphocytes are critical components of the adaptive immune system and have been shown to contribute to the development of hypertension [4]. These cells can infiltrate various organs, including the kidneys and vasculature, promoting inflammation and tissue remodeling [5]. Pro-inflammatory signaling molecules released from lymphocytes can alter vascular function and contribute to the chronic elevation of blood pressure [6].

There are three primary types of lymphocytes: T cells, B cells, and natural killer cells (NK cells). So far, only T and B cells have been reported to be involved with hypertension [7]. Angiotensin II (Ang II), a potent vasoconstrictor, can activate T-cell migration to target tissues [8]. Once there, T cells release pro-inflammatory cytokines such as interferon-gamma (IFN-γ) and tumor necrosis factor-alpha (TNF-α), which exacerbate vascular inflammation and oxidative stress [9]. This inflammatory milieu promotes endothelial dysfunction, characterized by impaired nitric oxide bioavailability and increased vascular stiffness, ultimately contributing to elevated blood pressure [10]. Moreover, regulatory T cells (Tregs), which generally suppress immune responses and maintain tolerance, are often dysfunctional in individuals with hypertension [11]. The imbalance between effector T cells and T regulatory cells (Tregs) can further amplify inflammation and vascular damage. B cells also play a role in hypertension by producing autoantibodies against the angiotensin II type 1 receptor (AT1R), thereby enhancing the effects of angiotensin II on vascular function and contributing to the development of hypertension [12]. Additionally, B cells can secrete pro-inflammatory cytokines, such as interleukin-6 (IL-6), which further stimulate T-cell activation and contribute to the inflammatory cascade [13]. These findings indicate that an interaction between B and T cells creates a feedback loop that perpetuates inflammation and hypertension.

While it is primarily associated with cardiovascular disease, recent research has suggested that hypertension reduces bone mass, density, and strength in the spontaneously hypertensive rat (SHR) model [14]. Bone metabolism is regulated by a balance between bone resorption and formation [15]. Osteoblasts are cells responsible for forming bone, while osteoclasts are cells responsible for resorbing bone. The balance between these two cell types is critical in maintaining bone health. RANKL (receptor activator of nuclear factor κB ligand), RANK (receptor activator of nuclear factor κB), and OPG (osteoprotegerin) are critical players in the regulation of bone resorption and formation. Osteoblasts produce RANKL that binds to RANK on the surface of osteoclast precursors, promoting their differentiation into mature osteoclasts. OPG is a decoy receptor produced by osteoblasts and binds to RANKL, preventing its interaction with RANK and thereby inhibiting osteoclastogenesis. The balance between RANKL and OPG is critical in regulating bone resorption and formation, and any disruption in this balance can lead to structural bone loss, such as in osteoporosis [16]. Bone structural and functional changes can be determined by micro-computed tomography (µ-CT) and a three-point bending mechanical test, respectively [17]. It has been demonstrated that bone health can be influenced by external factors, including physical force and nutrition [18].

Edible house crickets (*Acheta domesticus*) are nutritious, containing amino acids (especially sulfur-containing amino acids), fats, minerals, vitamins, and dietary fibers [19]. For example, sulfur-containing amino acids (methionine, cysteine, isoleucine, leucine, and lysine) have been shown to play essential roles in hypertension, utilizing their hydrogen sulfide molecule [20]. Previous studies have shown that house crickets contain chitin and its derivative, chitosan, which exhibit antioxidant and anti-inflammatory activity through modulation of T and B lymphocyte function [21–23]. Therefore, this study aimed to examine the effects of partially defatted cricket powder (PDCP) on bone microstructure and strength. We hypothesized that these experiments would provide insightful data that could help optimize the use of cricket extract to promote human health.

## Materials and Methods

### Laboratory animals and blood pressure measurements

Spontaneously hypertensive rats (SHR/KyoMlac or SHR; 19 weeks old) were acquired from the National Laboratory Animal Centre of Thailand (NLAC), Mahidol University. A non-invasive tail-cuff technique was used to assess blood pressure after a 5-day acclimatization period (CODA tail-cuff blood pressure apparatus, Kent Scientific Corporation, USA). The procedure entails holding the rats for five to ten minutes. Before the study, the animals were trained and acclimated by being kept in the holder for 15 minutes daily for three consecutive days [24]. In a 12-hour light-dark cycle with 30−70% relative humidity at 22 ± 3°C, fourteen hypertensive rats [systolic blood pressure (SBP) ≥ 140 mmHg] were randomly assigned to the experimental group (n = 7) and control group (n = 7) using a computer-generated random sequence to ensure baseline SBP was balanced. All animals had ad libitum access to a standardized diet containing 4000 IU/kg of vitamin D, 1.0% calcium, and 0.9% phosphorus, ensuring isocaloric conditions across groups (CP Co., Ltd., Thailand). Water treated by reverse osmosis was freely accessible. For four weeks, every rat in the experimental group received an oral gavage containing 300 mg/kg body weight (BW) of the PDCP diluted in a vehicle (1 milliliter of distilled water) once daily. This dose was determined using the body surface area normalization method to provide a human equivalent dose (HED) [25] of approximately 3 g/day for a 60 kg adult, aligning with realistic supplemental intake levels [26]. A previous acute toxicity study on *A. domesticus* powder showed no adverse effects at doses up to 3,000 mg/kg, confirming the safety of our selected 300 mg/kg dose [27]. The vehicle under control was given one milliliter of distilled water. The animals were euthanized using CO_2_ inhalation. To alleviate animal suffering, animals were kept warm with a blanket and heat lamp in a low-stress tranquil environment. The National Laboratory Animal Center Animal Care and Use Committee (NLAC-ACUC) authorized this study procedure (protocol approval number RA2023−16). The US National Institutes of Health’s Guide for the Care and Use of Laboratory Animals (updated 1996) [28] was followed in all operations.

### Preparation of partially defatted cricket powder

House crickets used in this study were obtained from Good Agricultural Practices (GAP)-certified farms. In Nam Phong, Khon Kaen province, Thailand, mature house crickets (45 days, 16–21 mm long, and 0.31–0.40 g body weight) were gathered from a GAP-cricket farm. After being cleaned with tap water and rinsed with distilled water, the insects were frozen at −20°C to kill them without starvation. Frozen crickets were mashed in a home compact blender (Food processor, Philips, Eindhoven, the Netherlands) and sieved through an 800 µm mesh screen. Afterward, the cricket powder was defatted using food-grade n-hexane in a 1:3 (w/v) ratio. To ensure the safety of the final product and the complete removal of residual solvent, the mixture was subjected to rotary evaporation, followed by drying in a thin-layer vacuum oven at 80 °C for 2 h until a constant weight was achieved. This protocol ensured that any remaining hexane was fully volatilized. During the 4-week feeding period, animals were monitored daily for behavioral signs of solvent toxicity, i.e., no adverse neurological symptoms or significant changes in liver enzymes (ALT/ALP) were observed, indicating that the PDCP was safe for consumption.

### Macronutrient analyses

A portion of 100 mg PDCP was combined with 10 ml of concentrated H_2_SO_4_ and a 5 g digestion mixture to obtain a clear solution. After allowing the digestion tube to reach room temperature, the contents were moved to a 100 ml volumetric flask. After mixing 20 ml of an aliquot with 50 ml of 1 N NaOH, the Kjeldahl distillation apparatus (Kjeltec™ 2100, Fisher Scientific, Pennsylvania, USA) was used to distill the mixture. After collecting the distillate in 25 milliliters of 4% H_3_BO_3_, it was titrated against 0.01 N HCl until the endpoint was reached. Following is how the percent nitrogen content (%N) was determined.


$$\begin{array}{c} Crude\;protein\;content=Percent\;nitrogen\;(\%\;N)\;\times \;\text{5}.\text{9}\text{5} \end{array}$$

$$\begin{array}{c} Percent\;nitrogen\;(\%\;N)=\frac{(X-Blank)\times \;\text{0}.\text{0}\text{0}\text{0}\text{1}\text{4}\;\times \;\text{1}\text{0}\text{0}}{Y} \end{array}$$

Where X= titer value (ml) and Y = sample weight (g).

Petroleum ether was used in the Soxhlet apparatus to measure total lipids. The Soxhlet apparatus was filled with a 2-g sample of defatted house cricket powder, and extraction was carried out for approximately 16 hours at a drip rate of 10 mL/min. The weight difference between before and after extraction was used to determine the total lipid content. The total lipid content was expressed as grams per 100 grams on a dry weight basis. Using in-house techniques (T974) based on AOAC procedures, the saturated fatty acid content of defatted cricket powder was determined [29]. The cholesterol content of defatted cricket powder was ascertained using internal T992-based techniques. After boiling the PDCP (2 g) in 250 ml of H_2_SO_4_ solution for 30 minutes, it was filtered through glass crucibles that had been sintered and carefully cleaned with hot water.

After 30 minutes of boiling the residual once again with 250 ml of 1 N NaOH, it was filtered and rinsed with hot water to produce a clear filtrate. A muffle furnace was used to dry and ash the residue at 600 °C. The crude fiber content was calculated and reported as a percentage based on the weight loss of the samples. After measuring every other component, the percentage of carbs that remained was calculated as follows: %carbohydrates = 100 (%moisture + %protein + %lipid + %ash). The following formula was used to estimate the gross food energy [30].


$$\begin{array}{c} Food\;energy\;(kCal/g)=(CP\;\times \;\text{4})+(F\;\times \;\text{9})+(CHO\times \text{4}) \end{array}$$

CP = crude protein (%), F = fat (%), and CHO = carbohydrate content (%).

### Micronutrient analyses

The in-house technique T9152, based on AOAC (2019) 984.27, was used to determine the concentrations of calcium, iron, magnesium, phosphorus, potassium, sodium, and zinc. In-house techniques T915 were used to analyze Selenium. In-house methods T969, T9670, T9671, and T9236 were used to determine vitamin A, B1, B2, and D, respectively, based on methods 992.06, 942.23, 970.65, and 995.05.

### Immune cell counts

BD Vacutainer™ glass tubes containing K3 EDTA (Thermo, MA, USA) were used to collect whole blood. According to the manufacturer’s instructions, immune cell tests were performed using an IDEXX ProCyte DX hematology analyzer (IDEXX, Westbrook, ME, USA). The output reports included counts and percentages of white blood cells, neutrophils, lymphocytes, monocytes, eosinophils, and basophils, among other immune cell types.

### Bone mechanical property testing

**Fig 1:**
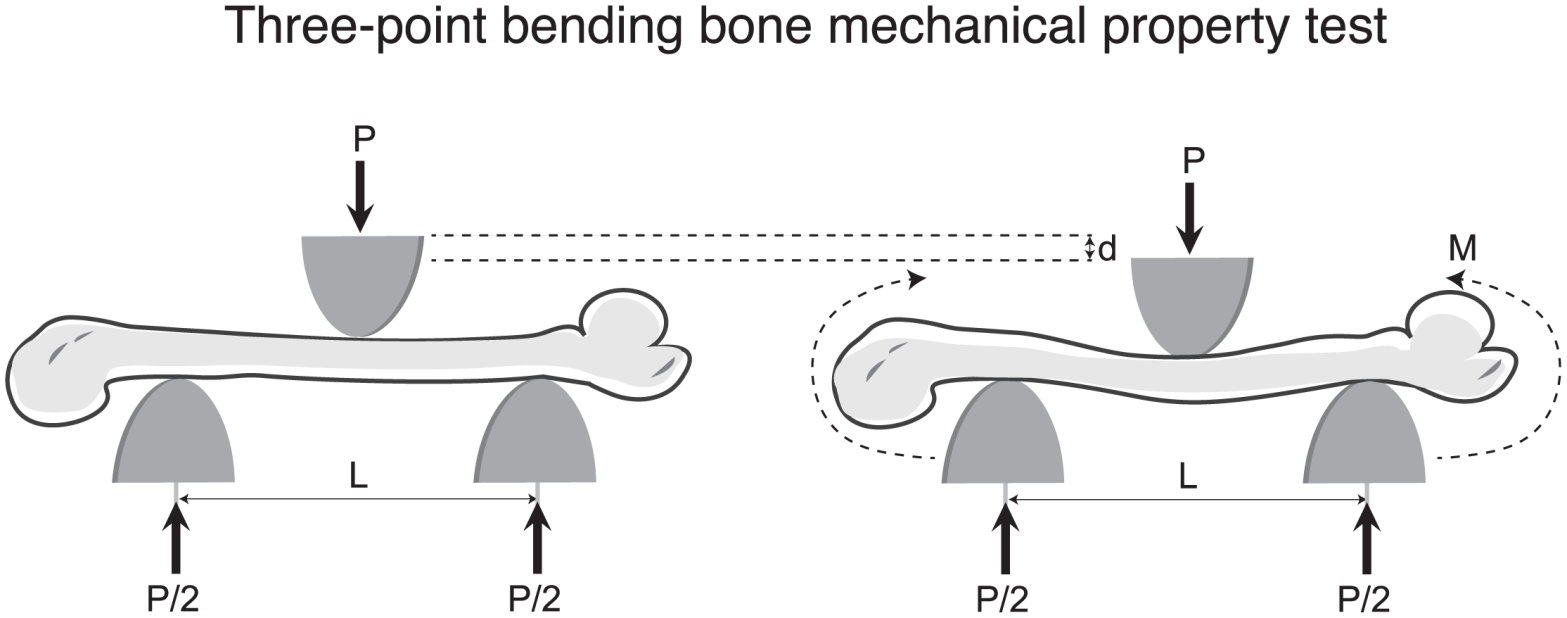
Schematic representation of a rat femur loaded in three-point bending bone mechanical property test. At the starting point (left), load (P) is applied to the bone surface in the middle of a width **(L)**. The transduced force results in the displacement (d) of the femoral shaft, dispersing to femoral ends (P/2) and generating moments (M) indicated by the curved arrows (right).

The flexional stiffness and strength of the femur were assessed using the three-point bending technique (Instron model 5943, Norwood, MA, USA). The femoral length and thickness at the mid-shaft were measured before each mechanical test. The crosshead displacement rate was 2 mm/min. The test was conducted on the mid-diaphysis of the femur, which was placed in two supports with a width (L) of 20 mm, with the femoral anterior edges facing downward toward the actuator (Fig 1). Load-displacement (P-D) curves for each specimen were generated using Instron5900 software (Norwood, MA, USA). Bone three-point bending parameters included maximum load, yield load, ultimate displacement, yield displacement, post-yield displacement, stiffness, energy absorption, flexure strain at break, flexure stress at break, flexure stress at maximum load, flexure strain at maximum load, and modulus, as previously described [31].

### Bone microarchitecture measurement

As previously mentioned, µCT (model 1178, SkyScan, Aartselaar, Belgium) was used to assess the volume of the femoral trabecular and cortical bone [32]. A 0.5-mm aluminum filter and a 65-kV X-ray tube with a 615 lA current were equipped in the apparatus. With a 0.54° angular increment, the scanning angular rotation was 180°. The proximal and distal growth plates of long bones (50 slides) were covered by the volume of interest (VOI), which varied from 1.43 to 1.86 mm. VOI for trabecular bone analysis was selected starting 1.0 mm distal to the lower end of the femoral growth plate and extending 2.0 mm further. This gap ensures that the primary spongiosa (highly dynamic bone) is excluded, focusing the analysis on the more stable secondary spongiosa. Reconstructing and processing the pictures was performed using a computer cluster using the SkyScan CT-analyzer software program (version 1.11.10).

### Statistical analysis

SPSS Statistics version 19.0 (Statistical Package for the Social Sciences; IBM) was used to conduct the statistical analysis. The mean ± standard error of the mean was used to express the results. The nonparametric Mann-Whitney U test and Wilcoxon signed-rank test were used to compare two means of independent and paired groups, respectively, at *P* < 0.05.

## Results

### Macronutrient and micronutrient contents in partially defatted cricket powder

The macronutrient and micronutrient contents in PDCP were analyzed and are presented in Table 1. Results showed that it contained 429 kcal/100 g of energy, 70.7 g/100 g of protein, 7.2 g/100 of carbohydrate, no sugar, and 13.0 g/100 of total fat. Dietary fiber was 7.2 g/100 g. Regarding minerals, PDCP has the highest amount of potassium, followed by phosphorus, sodium, calcium, and magnesium. Selenium, zinc, and iron were present in small fractions. Interestingly, vitamin B2 was the only one detected.

**Table 1:** Macronutrient and micronutrient contents in partially defatted house cricket (*Acheta domesticus*) powder.

Parameter	Unit	Result	LOD	LOQ
**Macronutrient**				
Total energy	kcal/100 g	429.0	N/A	N/A
Total protein	g/100 g	70.7	0.1	0.3
Total carbohydrate	g/100 g	7.2	N/A	N/A
Total sugar	g/100 g	N/D	N/A	N/A
Total fat	g/100 g	13.0	N/A	N/A
Cholesterol	g/100 g	0.2	0.15	0.3
Saturated fatty acid	g/100 g	4.3	0.02	0.05
Dietary fiber	g/100 g	7.2	N/A	N/A
**Micronutrient**				
*Mineral*				
Calcium	mg/100 g	183.0	0.6	10.7
Iron	mg/100 g	6.5	0.007	0.243
Magnesium	mg/100 g	138.0	0.010	0.070
Phosphorus	mg/100 g	976.0	0.037	1.536
Potassium	mg/100 g	1,046.0	0.7	10.2
Sodium	mg/100 g	387.0	2.8	10.0
Selenium	mg/100 g	42.6	1.0	2.0
Zinc	mg/100 g	25.1	0.003	0.072
*Vitamin*				
Vitamin A	µg/100 g	N/D	20.0	N/A
Vitamin B1	mg/100 g	N/D	0.002	0.004
Vitamin B2	mg/100 g	1.66	0.004	0.008
Vitamin D	µg/100 g	N/D	2.0	N/A

LOD, limit of detection; LOQ, limit of quantitation; N/A, not applicable; N/D, not detected.

### Antihypertensive activity of the partially defatted house cricket powder

**Fig 2:**
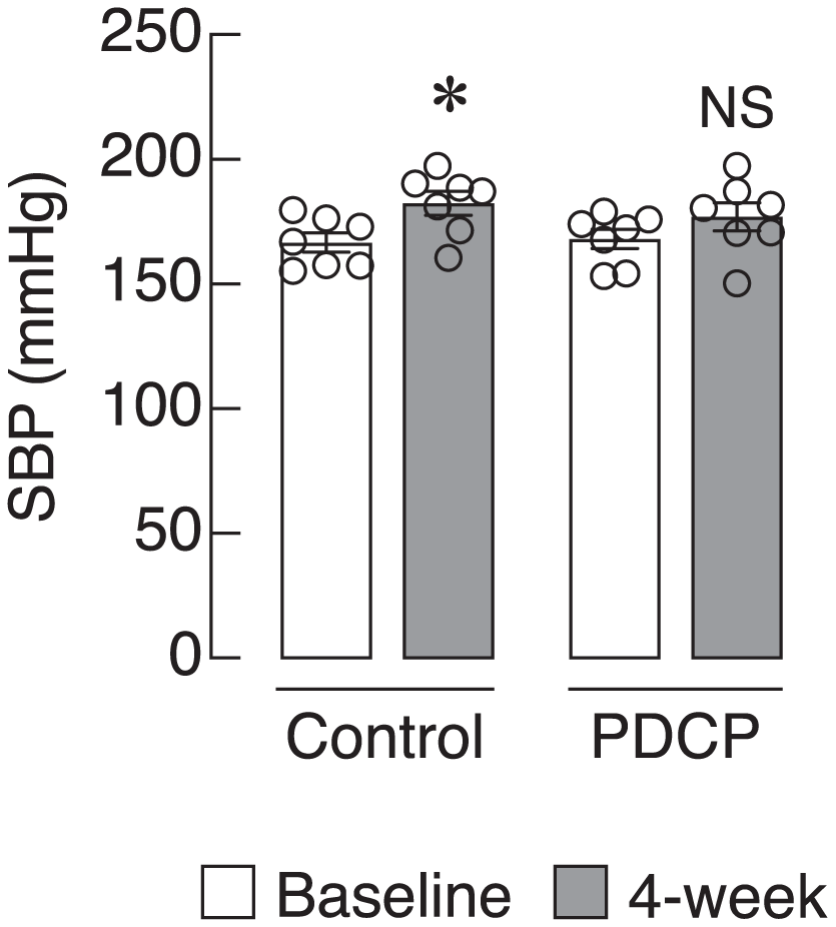
Effect of partially defatted cricket powder (PDCP) on systolic blood pressure (SBP) of spontaneously hypertensive rats (SHRs). A, baseline group, and 4-week follow-up (n = 7). B, baseline cricket (PDCP)-treated group and 4-week post-intervention (n = 7). *, significantly different at *P* < 0.05. NS is not significantly different.

Results showed that SBP baselines in the control and PDCP-treated groups were 166.6 ± 3.7 and 168.1 ± 3.9 mmHg, respectively (*P* = 0.79). After four weeks of interventions, the SBP in the control group significantly increased to 182.4 ± 4.7 mmHg (*P* = 0.016). In contrast, the SBP in the PDCP-treated group, 176.9 ± 5.2 mmHg, did not significantly alter when compared to baseline (*P* = 0.156) (Fig 2). This prompted us to investigate further the effects of PDCP on bone microarchitecture and strength.

### Partially defatted house cricket (*Acheta domesticus*) powder did not alter bone microarchitecture

**Fig 3:**
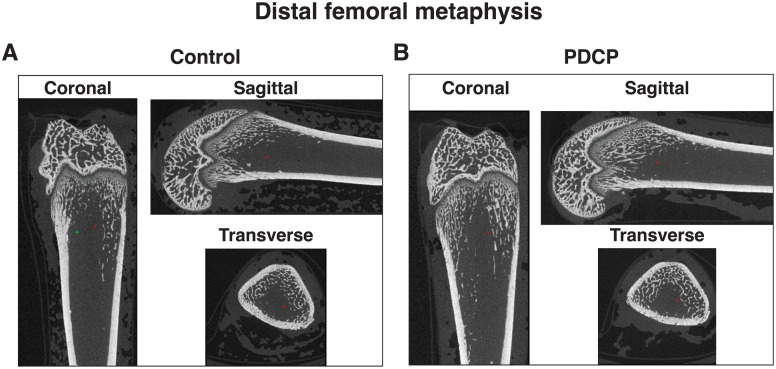
Representative µCT images of distal femora from female SHRs. Two-dimensional µCT slices of the distal femoral metaphysis illustrate trabecular microarchitecture following a 4-week intervention. Images depict the control group (A) in the transverse, coronal, and sagittal planes, and the PDCP-treated group (B) in the transverse, coronal, and sagittal planes.

Table 2 shows that the cortical volume/total volume, trabecular thickness, trabecular separation, bone mineral density, and bone mineral content of femoral bones in control and PDCP-treated groups were not significantly different (*P* = 1.000, 0.383, 0.902, 0.565, and 1.000, respectively). Fig 3 shows representative 2D µCT slices of the distal femoral metaphysis to illustrate the trabecular microarchitecture typical of both the control and PDCP-treated groups.

**Table 2:** Effects of partially defatted house cricket (*Acheta domesticus*) powder on bone microarchitecture.

Parameter	Control	Cricket powder	*P*-value
Cortical volume/Total volume (%)	5.53 ± 0.29	5.73 ± 0.31	1.000
Trabecular thickness (mm)	92.77 ± 0.97	91.44 ± 0.61	0.383
Trabecular separation (mm)	212.17 ± 7.46	201.94 ± 11.21	0.902
Volumetric bone mineral density (g/cm^3^)	0.121 ± 0.003	0.122 ± 0.003	0.565
Bone mineral content (mg)	1.565 ± 0.060	1.576 ± 0.064	1.000

### Partially defatted house cricket (*Acheta domesticus*) powder improved bone strength

The yield load bone three-point bending parameter in the PDCP group was significantly higher than in the control (83.58 ± 1.26 vs 77.55 ± 1.88 N). Moreover, yield displacement was also higher (395.93 ± 40.98 vs 307.72 ± 29.78 µm). The remaining parameters (maximum load, ultimate displacement, post-yield displacement, stiffness, energy absorption, flexure strain at break, flexure stress at break, flexure stress at maximum load, flexure strain at maximum load, and modulus) were not statistically different (Table 3).

**Table 3:** Effects of partially defatted house cricket (*Acheta domesticus*) powder on bone structural mechanical properties.

Parameters	Control	Cricket powder	*P*-value
Femoral length (mm)	31.49 ± 0.13	31.62 ± 0.10	0.310
Maximum load (N)	102.93 ± 1.46	103.07 ± 1.48	0.355
Yield load (N)	77.55 ± 1.88	83.58 ± 1.26	0.009*
Ultimate displacement (µm)	553.96 ± 22.35	622.49 ± 47.38	0.267
Yield displacement (µm)	307.72 ± 29.78	395.93 ± 40.98	0.049*
Post-yield displacement (µm)	246.24 ± 20.46	226.55 ± 24.61	0.841
Stiffness (N/mm)	342.62 ± 10.01	340.85 ± 8.28	0.841
Energy absorption (N-mm)	32.77 ± 1.621	33.85 ± 2.41	0.355
Flexure strain at break (mm/mm)	0.031 ± 0.001	0.036 ± 0.003	0.067
Flexure stress at break (MPa)	264.53 ± 3.87	265.40 ± 3.87	0.355
Flexure stress at maximum load (MPa)	268.42 ± 3.81	268.773 ± 3.88	0.355
Flexure strain at maximum load (mm/mm)	0.031 ± 0.001	0.036 ± 0.003	0.067
Modulus (MPa)	14980.7 ± 516.4	14660.1 ± 442.8	0.809

### Partially defatted house cricket (*Acheta domesticus*) powder did not affect renal and liver functions

As the kidneys and liver are vital to bone metabolism and mineral homeostasis, we investigated the effects of PDCP on these organ systems [33]. The results demonstrated that while several parameters—including serum potassium (K^+^), AST, and the BUN/Creatinine ratio—were apparently higher than standard reference ranges in both the control and PDCP-treated groups, there were no significant differences between the two groups (*P* > 0.05; Table 4). It is noteworthy that the pseudohyperkalemia observed in both groups may plausibly be attributed to a technical limitation, namely, blood sampling through a relatively small needle. None of the animals manifested signs of hyperkalemia, including lethargy, muscle weakness, reduced activity, and abnormal respiratory patterns.

**Table 4:** Effects of partially defatted house cricket (*Acheta domesticus*) powder on liver and kidney functions.

Parameter	Reference range	Control	Cricket powder	*P*-value
*Liver function*				
Aspartate aminotransferase (U/L)	121.95 ± 5.16	165.1 ± 23.4	222.5 ± 33.0	0.609
Alanine aminotransferase (U/L)	29.66 ± 1.94	66.0 ± 9.3	64.3 ± 4.3	0.805
Alkaline phosphatase (U/L)	113.79 ± 4.73	137.4 ± 12.8	141.4 ± 8.9	0.902
*Kidney function*				
Blood urea nitrogen (mg/dL)	39.02 ± 1.74	18.9 ± 0.8	18.5 ± 0.9	0.620
Creatinine (mg/dL)	0.54 ± 0.02	0.226 ± 0.003	0.227 ± 0.006	0.947
Blood urea nitrogen/Creatinine ratio	18.75 to 55	84.2 ± 4.2	81.7 ± 3.3	0.805
Na^+^ (mEq/L)	135–150	147.0 ± 0.3	147.8 ± 0.4	0.185
K^+^ (mEq/L)	3.5–5.5	7.27 ± 0.12	7.04 ± 0.24	0.456
Cl^–^ (mEq/L)	95–110	108.3 ± 0.3	108.0 ± 0.1	0.564

Reference ranges are based on data from He et al. [34], using the upper and lower limits of the 90% confidence interval.

### Partially defatted house cricket (*Acheta domesticus*) powder altered immune cell expression

As inflammation, hypertension, and osteoporosis are interrelated [35], we examined the immune cell counts in control and PDCP-treated spontaneously hypertensive rats (SHRs). The results showed that total white blood cell and lymphocyte counts were significantly increased in the PDCP group (Table 5).

**Table 5:** Effects of partially defatted house cricket (*Acheta domesticus*) powder on immune cell counts.

Parameter	Reference range	Control	Cricket powder	*P*-value
White blood cell (10^3^/µL)	2.40–7.96	6.91 ± 0.31	7.91 ± 0.272	0.017*
Neutrophil (10^3^/µL)	0.15–1.07	0.75 ± 0.04	0.78 ± 0.04	0.449
Lymphocyte (10^3^/µL)	1.83–6.59	5.75 ± 0.28	6.66 ± 0.22	0.017*
Monocyte (10^3^/µL)	0.04–0.45	0.33 ± 0.02	0.38 ± 0.04	0.282
Eosinophil (10^3^/µL)	0.03–0.25	0.05 ± 0.00	0.06 ± 0.01	0.500
Basophil (10^3^/µL)	0–0.02	0.01 ± 0.00	0.01 ± 0.00	0.803
% Neutrophil	3.84–25.94	10.91 ± 0.62	9.94 ± 0.47	0.875
% Lymphocyte	61.24–91.74	83.20 ± 0.73	84.22 ± 0.56	0.221
% Monocyte	0.94–10.64	4.84 ± 0.27	4.87 ± 0.48	0.649
% Eosinophil	0.64–6.84	0.80 ± 0.07	0.80 ± 0.16	0.761
% Basophil	0.04–0.64	0.24 ± 0.08	0.157 ± 0.57	0.804

Reference ranges are based on data from He et al. [34], using the upper and lower limits of the 90% confidence interval. *P < 0.05 vs. Control.

## Discussion

As hypertension negatively affects bone metabolism, this study investigated the effects of the partially defatted cricket powder (PDCP) on SBP and bone microarchitecture and strength. We found that the PDCP attenuated the increase in SBP and improved bone strength but did not affect bone microarchitecture. Below, we discuss the possible mechanisms involved in the positive effects of PDCP on blood pressure and bone strength, including aspects related to hormones and cytokines.

Protein intake is positively associated with bone strength, as a previous study showed that protein intake reduced the risk of hip fracture in postmenopausal adults [36]. Dietary protein is a known stimulator of insulin-like growth factor I (IGF-I) synthesis, an anabolic factor essential for bone trophic support [37]. While IGF-1 levels were not quantified in the present study, the high protein content of our PDCP (75.34 g/100 g) aligns with levels previously associated with reduced fracture risk [36]. It is hypothesized that the observed improvements in bone strength may be partially mediated by protein-induced anabolic signaling, though further endocrine profiling is required to confirm this pathway. Additionally, total carbohydrate and fat contents were higher than previously reported, whereas the dietary contents were similar (7.2 vs. 7.7 g/100 g) [38].

According to collective research, the dietary fiber (chitin and lignin) contents of edible insects range from 5 to 15% [39]. Chitin possesses recognized prebiotic properties [26]. While gut microbiota analysis was outside the scope of this study, previous research has shown that cricket powder can increase *Bifidobacterium animalis*, which may inhibit osteoclastogenesis [40]. Therefore, the high fiber levels in PDCP represent a potential prebiotic mechanism that might support bone mechanical strength. Further study is essential to verify the relationship between insect-derived fiber and the gut-bone axis.

We found increased bone strength as indicated by yield load and displacement. Yield load is the maximal load supported by bones in linear elasticity before any crack initiation within the mineralized tissue [41]. Bone strength relies on two phases of materials: the inorganic phase, consisting of minerals, and the organic phase, comprised of collagen fibers [42]. At the microscopic level, biomechanical bone quality depends on the distribution and alignment of minerals and collagen [43]. In contrast, at the nano level, it depends on collagen structure and cross-linking, mineral type and crystal alignment, collagen-mineral interfaces, and type, amount, and distribution of microdamage [43]. Interestingly, a previous study reported increased whole-body bone mineral content (BMC) in a house cricket-fed, malnourished rat model [44].

As no changes in BMC were observed, the increase in bone strength is unlikely to be attributable to additional mineralization. Instead, we propose that an improvement in the intrinsic material properties of the bone matrix may underlie this effect [45]. We also hypothesize that copper as a critical cofactor for lysyl oxidase-mediated cross-linking, together with collagen-precursor amino acids (proline and glycine) found in *A. domesticus*, may have contributed to enhanced collagen matrix stabilization [46]. Furthermore, cricket-derived peptides have demonstrated antioxidant activities in other models, which could theoretically mitigate the formation of brittle nonenzymatic cross-links [47]. In the absence of Raman spectroscopy or nanoindentation data, these nanostructural modifications remain speculative but may represent a plausible explanation for the observed improvement in yield load.

Regarding bone physiological changes, Ganguly and colleagues showed that two-spotted cricket (*Gryllus bimaculatus*) protein isolate (CPI) induced an *in vitro* osteogenic differentiation of human bone marrow-derived mesenchymal stem cells (hBMSCs) as signified by osteoinductive genes [Runt-related transcription factor 2 (*Runx2*), alkaline phosphatase (*ALP*), osteocalcin (*OCN*), bone sialoprotein (*BSP*), and collagen type 1 (*COL1*)] [48]. Moreover, they also found that CPI stimulated mineralization in hBMSCs. In the present study, it can be inferred that collagen cross-linking and realignment may occur. Further extensive studies are still needed to understand this issue thoroughly.

Micronutrient constituents in the PDCP play a pivotal role in bone metabolism. Calcium and phosphorus are major components of bone hydroxyapatite, while sodium and potassium play essential roles in maintaining the acid-base balance of the bone surface [33,49]. Additionally, magnesium serves as a cofactor for vitamin D, maintaining a balance between bone formation and resorption [50]. Selenium is essential for the synthesis of selenoproteins, which play a crucial role in bone growth and development [51]. Zinc regulates bone metabolism through at least three mechanisms: facilitating bone remodeling by controlling the RANKL/RANK/OPG pathway, promoting osteoblast differentiation, and inducing osteoclast apoptosis, thereby resulting in positive bone formation [52]. Vitamin B2 has been found to induce osteoblast differentiation and, when combined with hydroxyapatite, to enhance bone tissue regeneration [53]. These micronutrients in PDCP might orchestrate positive signals to preserve bone strength under high blood pressure conditions.

Regarding the effects of PDCP on bone and blood pressure, a limitation of the present study is the 4-week intervention period. In adult rats, the bone remodeling cycle typically spans approximately 3–4 weeks. Therefore, the study duration may have been insufficient to capture significant changes in bone microarchitecture or volume as assessed by µCT [45]. Furthermore, the absence of biochemical markers of bone turnover (e.g., P1NP and CTX-1) limits our ability to quantify rates of bone formation and resorption. However, the observed improvement in mechanical strength within this timeframe suggests that PDCP may exert rapid effects on the intrinsic material quality of the bone matrix, potentially preceding long-term structural changes. A previous study found that feeding silkworm (*Bombyx mori*) protein hydrolysates for 4 weeks reduced SBP in SHRs [54]. More recently, Pessina and colleagues reported that feeding SHRs defatted mealworm larvae (*Tenebrio molitor; Coleoptera: Tenebrionidae*) for 4 weeks reversed to a basal level, comparable to the effect of an antihypertensive drug [55]. Mechanistically, it has been claimed that the silkworm and mealworm diets inhibited a blood pressure-stimulatory enzyme, angiotensin-converting enzyme (ACE). Recently, peptides produced during *in vitro* gastrointestinal digestion of *A. domesticus* have been reported to inhibit ACE activity *in silico* [56]. This finding might imply the role of the ACE inhibitory effect of the *A. domesticus*-derived PDCP in the present study.

In addition to angiotensin II, certain inflammatory cytokines play pivotal roles in the progression of hypertension and osteoporosis [35]. A low relative lymphocyte count was associated with an adverse clinical profile in patients with heart disease, including a lower ejection fraction, higher natriuretic peptide levels, and elevated blood pressure [57]. Osteopenic and osteoporotic patients had lower lymphocyte counts than healthy individuals [35]. The hematological analysis revealed increased total WBC and lymphocyte counts in the cricket-supplemented group. While such increases can be indicative of immune stimulation or systemic inflammation, the concurrent attenuation of blood pressure elevation suggests a non-pathogenic, immunomodulatory effect.

Hypertension is increasingly recognized as resulting from an imbalance between regulatory T cells (Tregs) and T helper 17 (Th17) cells. While Tregs exert a protective, homeostatic influence by inhibiting angiotensin II-mediated vascular damage [58], Th17 cells actively facilitate angiotensin II-induced hypertensive progression through the promotion of systemic inflammation and vascular remodeling [59]. Although we did not perform flow cytometry to assess the Tregs/Th17 ratio, the osteoprotective nature of this immune shift is inferred from the concurrent improvement in functional bone and vascular parameters. The concurrent increases in total WBC and lymphocyte counts present a lymphocyte paradox in hypertension, where immune activation can be either pathogenic or protective [60].

While elevated lymphocytes are traditionally markers of systemic inflammation that drives bone resorption, the simultaneous attenuation of the SBP rise in our study suggests a state of immune-vascular homeostasis [58]. This state likely involves the expansion of Tregs that suppress osteoclast formation and protect the bone matrix [61]. In the absence of biochemical bone turnover markers (e.g., P1NP and CTX-1) or direct osteoclast activity assays, the specific molecular pathways–whether mediated by suppressed osteoclastogenesis or enhanced osteoblast function–cannot be definitively identified. We hypothesize that the unique nutritional matrix of *A. domesticus* may act through an integrative immune-vascular axis, but this remains a speculative framework.

We hypothesize that the prebiotic-like action of insect chitin may facilitate a shift toward a homeostatic immune surveillance profile [62]. However, as specific markers for allergenicity (e.g., IgE) and regulatory T-cell subsets were not quantified, these findings remain exploratory. Future studies should focus on the Th1/Th2/Treg balance to elucidate the precise mechanistic link between insect-derived nutrients and vascular immune-homeostasis.

The kidneys and liver are significant organs regulating bone metabolism [33]. A hydroxylase enzyme in the liver converts vitamin D to 25-hydroxyvitamin D, which will be further converted to active 1,25-hydroxyvitamin D by 1α-hydroxylase enzyme in the renal proximal tubule [63]. Moreover, upon being stimulated by 1,25-hydroxyvitamin D, renal tubules actively reabsorb calcium, an essential mineral component of bone [64]. Therefore, we examined the effects of PDCP on liver and kidney functions. The levels of liver and kidney parameters indicate kidney injury (as evidenced by blood urea nitrogen and creatinine) and liver injury (as indicated by aspartate aminotransferase, alanine aminotransferase, and alkaline phosphatase) [65,66]. However, we did observe the modulatory effects of PDCP on liver and kidney functions. The elevated serum K^+^ (approximately 7 mEq/L) across all groups could be attributed to pseudohyperkalemia resulting from partial hemolysis during microsampling, as subjects exhibited no apparent clinical or cardiovascular distress. Furthermore, since ALT and ALP remained within normal ranges, the elevated AST may reflect systemic metabolic activity rather than hepatocellular injury. These findings suggest that a 4-week intervention with PDCP did not induce acute systemic toxicity or impair renal or liver function compared with the control group [67,68].

A limitation of this study is the inability to perform longitudinal skeletal assessments. Future studies could employ *in vivo* µCT (scanning live rats under anesthesia at week 0 and week 4) to provide within-group architectural comparisons. However, our use of age-matched, randomized SHRs with identical baseline weights and SBP minimizes the likelihood of pre-existing skeletal variability.

## Conclusion

This study demonstrates that partially defatted cricket powder (PDCP) attenuates the progression of SBP and enhances intrinsic bone quality in SHRs. The observed increase in lymphocyte counts, occurring alongside stabilized SBP, suggests a state of immune-vascular homeostasis rather than pathogenic inflammation. This shift may reflect an expansion of regulatory T-cell (Treg) populations or an overall improvement in immune vigor, which could, in turn, suppress osteoclastogenesis. Furthermore, the improvement in mechanical yield load, in the absence of microarchitectural changes, suggests stabilization of the mineral-to-collagen matrix. While the observed effects may be attributed to bioactive peptides (ACE-inhibitory) and chitin/fibers (prebiotic-immunomodulatory), the specific roles of IGF-1 and modulation of the gut-bone axis require further molecular validation.

## Supporting information

S1 File Supplementary Information – Raw data. (PDF)
