# Supplementary material for: Osteoprotective effects of partially defatted house cricket (Acheta domesticus) powder in spontaneously hypertensive rats
Source: PLoS One. 2026 May 18;21(5):e0349511. doi: 10.1371/journal.pone.0349511 (PMC13183235; doi:10.1371/journal.pone.0349511)
Supplement: S1 File — (PDF) [file pone.0349511.s001.pdf]

Osteoprotective effects of partially defatted house cricket (*Acheta domesticus*) powder in spontaneously hypertensive rats

Kukiat Tudpor [1,2], Kasama Wongprachum [1,2], Tarinee Nilkamheang [1,2], Chaloemporn Namyota [1,2], Nitchara Toontom [1,2], Le Ke Nghiep [3], Khuanjit Chaimongkolnukul [4], Surachai Chantip [4], Nontawan Choovattanapakorn [4], Panan Suntornsaratoon [5,6], Kannikar Wongdee [5,10], Jarinthorn Teerapornpuntakit [5,11], Narattaphol Charoenphandhu [5,6,7,8]*, Sirithon Siriamornpun [9,**]

[1] Faculty of Public Health, Mahasarakham University, Maha Sarakham, Thailand
[2] Public Health and Environmental Policy in Southeast Asia Research Cluster (PHEP-SEA) and Faculty of Public Health, Mahasarakham University, Maha Sarakham, Thailand
[3] Vinh Long Department of Health, Vietnam
[4] National Laboratory Animal Center, Mahidol University, Nakhon Pathom, Thailand
[5] Center of Calcium and Bone Research (COCAB), Faculty of Science, Mahidol University, Bangkok, Thailand
[6] Department of Physiology, Faculty of Science, Mahidol University, Bangkok, Thailand
[7] Institute of Molecular Biosciences, Mahidol University, Nakhon Pathom, Thailand
[8] The Academy of Science, The Royal Society of Thailand, Bangkok, Thailand
[9] Research Unit of Thai Food Innovation, Department of Food Technology and Nutrition, Mahasarakham University, Maha Sarakham, Thailand
[10] Faculty of Allied Health Sciences, Burapha University, Chonburi, Thailand
[11] Physiology Division, Preclinical Science, Faculty of Medicine, Thammasat University, Pathum Thani, Thailand

Short title: Osteoprotective effects of cricket powder in hypertensive rats

Keywords: anti-inflammation; bone strength; edible insects; hypertension; micro-computed tomography

*** Corresponding author**

Narattaphol Charoenphandhu, M.D., Ph.D.
Department of Physiology, Faculty of Science, Mahidol University
Rama VI Road, Bangkok, 10400, Thailand
E-mail: narattaphol.cha@mahidol.ac.th

**** Co-corresponding author**

Sirithon Siriamornpun, Ph.D.
Department of Food Technology and Nutrition, Faculty of Technology,
Mahasarakham University, Kham Riang, Kantharawichai, Maha Sarakham, 44150, Thailand
E-mail: sirithon.s@msu.ac.th

Figure 2. Effect of partially defatted cricket powder (PDCP) on systolic blood pressure (SBP) of spontaneously hypertensive rats (SHRs).

	Baseline control	4-week control	Mean difference	Baseline cricket	4-week cricket	Mean difference
	155.30	160.60	5.30	172.30	181.70	9.40
	157.40	190.30	32.90	179.10	180.50	1.40
	166.90	181.10	14.20	154.20	150.30	-3.90
	173.10	197.40	24.30	167.80	197.30	29.50
	176.40	187.20	10.80	153.30	170.80	17.50
	179.70	188.70	9.00	174.10	187.20	13.10
	157.70	171.50	13.80	176.00	170.60	-5.40
Mean	166.64	182.40	15.76	168.11	176.91	8.80
SD	9.27	11.62	9.60	9.63	13.85	12.52
SEM	3.50	4.39	3.63	3.64	5.23	4.73

Table 2. Effects of partially defatted house cricket (*Acheta domesticus*) powder on bone microarchitecture

Group	Cortical volume/Total volume (%)	Trabecular thickness (mm)	Trabecular separation (mm)	Volumetric bone mineral density (g/cm^3)	Bone mineral content (mg)
Control	6.118	91.000	206.688	0.123	1.635
Control	4.515	89.860	228.474	0.107	1.355
Control	6.970	90.260	171.781	0.132	1.563
Control	5.428	94.400	211.715	0.122	1.616
Control	5.218	94.490	221.158	0.118	1.365
Control	5.385	96.810	230.208	0.124	1.799
Control	5.116	92.620	215.230	0.118	1.624
Mean	5.536	92.777	212.179	0.121	1.565
SD	0.732	2.386	18.295	0.007	0.146
SEM	0.277	0.902	6.915	0.003	0.055
Cricket powder	4.850	92.150	235.168	0.121	1.326
Cricket powder	6.969	89.410	171.876	0.128	1.617
Cricket powder	5.000	94.410	238.623	0.110	1.513
Cricket powder	5.967	90.830	177.187	0.126	1.793
Cricket powder	5.061	91.470	221.972	0.118	1.417
Cricket powder	5.678	91.740	196.585	0.125	1.619
Cricket powder	6.618	90.070	172.179	0.129	1.750
Mean	5.735	91.440	201.941	0.122	1.576
SD	0.769	1.502	27.469	0.006	0.157
SEM	0.291	0.568	10.382	0.002	0.059

Table 3. Effects of partially defatted house cricket (*Acheta domesticus*) powder on bone structural mechanical properties

Group	Femoral length (mm)	Maximum load (N)	Yield load (N)	Ultimate displacement (µm)	Yield displacement (µm)	Post-yield displacement (µm)
Control	31.26	100.63	79.32	530.04	329.85	200.19
Control	31.42	104.58	72.19	563.30	230.28	333.02
Control	31.48	99.65	79.21	446.44	236.59	209.85
Control	31.88	109.73	82.06	569.10	305.70	263.40
Control	30.88	105.70	69.15	576.49	292.99	283.50
Control	31.82	99.24	82.37	643.40	466.60	176.80
Control	31.71	101.04	78.56	549.00	292.04	256.96
Mean	31.49	102.94	77.55	553.97	307.72	246.25
SD	0.32	3.58	4.63	54.75	72.95	50.14
SEM	0.12	1.35	1.75	20.69	27.57	18.95
Cricket powder	31.55	96.76	82.40	519.90	333.05	186.85
Cricket powder	31.14	106.03	81.00	739.52	379.74	359.78
Cricket powder	31.65	101.47	84.15	526.03	298.98	227.05
Cricket powder	32.00	102.96	80.73	832.34	586.48	245.86
Cricket powder	31.56	103.03	90.22	649.79	479.77	170.02
Cricket powder	31.66	109.43	81.50	500.37	280.62	219.75
Cricket powder	31.80	101.82	85.10	589.48	412.92	176.56
Mean	31.62	103.07	83.59	622.49	395.94	226.55
SD	0.24	3.65	3.10	116.07	100.38	60.29
SEM	0.09	1.38	1.17	43.87	37.94	22.79

Table 3. (cont.)

Group	Stiffness (N/mm)	Energy absorption (N-mm)	Flexure strain at break (mm/mm)	Flexure stress at break (MPa)	Flexure stress at maximum load (MPa)	Flexure strain at maximum load (mm/mm)	Modulus (MPa)
Control	345.65	28.58	0.03	262.41	262.41	0.03	15351.53
Control	346.45	38.97	0.03	257.56	272.70	0.03	15288.50
Control	367.51	28.05	0.03	259.84	259.84	0.03	16377.18
Control	367.75	35.64	0.03	286.09	286.13	0.03	16326.39
Control	288.63	35.15	0.03	268.71	275.63	0.03	12380.99
Control	338.48	28.77	0.04	258.09	258.77	0.04	14332.43
Control	343.88	34.27	0.03	259.01	263.48	0.03	14808.19
Mean	342.62	32.78	0.03	264.53	268.42	0.03	14980.74
SD	24.52	3.97	0.00	9.48	9.34	0.00	1265.01
SEM	9.27	1.50	0.00	3.58	3.53	0.00	478.13
Cricket powder	325.58	29.07	0.03	251.85	252.31	0.03	14510.69
Cricket powder	315.53	47.19	0.04	258.51	276.49	0.04	13194.08
Cricket powder	340.81	32.65	0.03	264.60	264.60	0.03	14953.80
Cricket powder	332.87	36.47	0.05	263.74	268.48	0.05	14093.06
Cricket powder	342.19	31.55	0.04	268.59	268.67	0.04	13941.00
Cricket powder	384.82	30.98	0.03	285.01	285.35	0.03	16897.09
Cricket powder	344.19	29.06	0.03	265.51	265.51	0.03	15031.43
Mean	340.85	33.85	0.04	265.40	268.77	0.04	14660.16
SD	20.30	5.92	0.01	9.48	9.52	0.01	1084.86
SEM	7.67	2.24	0.00	3.58	3.60	0.00	410.04

Table 4. Effects of partially defatted house cricket (*Acheta domesticus*) powder on liver and kidney functions

Group	AST	ALT	ALP	BUN	Cr	BUN/Cr ratio	Na	K	Cl
Control	144.5	62.3	152.0	19.7	0.2	85.7	146.0	7.2	108.2
Control	155.1	48.7	139.0	17.6	0.2	80.0	146.0	7.6	108.0
Control	95.6	47.7	172.0	23.0	0.2	104.6	147.0	7.4	107.2
Control	134.0	44.7	126.0	20.3	0.2	88.3	148.0	7.1	108.6
Control	293.9	112.3	101.0	19.2	0.2	87.3	147.0	6.7	107.4
Control	177.6	60.8	91.0	17.3	0.2	72.1	148.0	7.2	110.2
Control	155.1	85.7	181.0	15.8	0.2	71.8	147.0	7.7	108.6
Mean	165.1	66.0	137.4	19.0	0.2	84.2	147.0	7.3	108.3
SD	57.5	22.9	31.4	2.2	0.0	10.4	0.8	0.3	0.9
SEM	21.7	8.6	11.9	0.8	0.0	3.9	0.3	0.1	0.3
Experimental	133.6	53.6	184.0	22.7	0.2	94.6	148.0	7.3	107.3
Experimental	125.7	59.3	148.0	21.1	0.3	84.4	149.0	5.8	108.5
Experimental	133.5	52.6	138.0	17.4	0.2	75.7	146.0	7.9	107.9
Experimental	302.9	66.6	153.0	17.5	0.2	83.3	147.0	7.2	107.8
Experimental	317.8	82.3	120.0	19.0	0.2	90.5	149.0	6.9	107.9
Experimental	279.7	58.8	135.0	15.7	0.2	71.4	149.0	7.0	108.0
Experimental	264.4	77.2	112.0	16.7	0.2	72.6	147.0	7.1	108.7
Mean	222.5	64.3	141.4	18.6	0.2	81.8	147.9	7.0	108.0
SD	80.9	10.7	21.9	2.3	0.0	8.3	1.1	0.6	0.4
SEM	30.6	4.0	8.3	0.9	0.0	3.1	0.4	0.2	0.2

Table 5. Effects of partially defatted house cricket (*Acheta domesticus*) powder on immune cell counts

Group	WBC	NEU	LYMPH	MONO	EO	BASO	%NEU	%LYMPH	%MONO	%EO	%BASO
Control	6.19	0.76	4.99	0.37	0.06	0.01	12.2	80.6	6	1	0.2
Control	7.22	0.78	6.06	0.33	0.04	0.01	10.8	83.9	4.6	0.6	0.1
Control	6.88	0.58	5.9	0.32	0.06	0.02	8.3	85.8	4.7	0.9	0.3
Control	8.03	0.75	6.77	0.38	0.07	0.06	9.4	84.3	4.7	0.9	0.7
Control	5.5	0.64	4.56	0.25	0.04	0.01	11.7	82.9	4.5	0.7	0.2
Control	7.15	0.78	6.02	0.27	0.07	0.01	10.9	84.2	3.8	1	0.1
Control	7.45	0.97	6.01	0.42	0.04	0.01	13.1	80.7	5.6	0.5	0.1
Mean	6.9	0.8	5.8	0.3	0.1	0.0	10.9	83.2	4.8	0.8	0.2
SD	0.8	0.1	0.7	0.1	0.0	0.0	1.5	1.8	0.7	0.2	0.2
SEM	0.3	0.0	0.3	0.0	0.0	0.0	0.6	0.7	0.3	0.1	0.1
Experimental	8.24	0.76	6.86	0.55	0.06	0.01	9.2	83.3	6.7	0.7	0.1
Experimental	7.45	0.67	6.4	0.29	0.06	0.03	9	85.9	3.9	0.8	0.4
Experimental	7.72	0.66	6.67	0.32	0.05	0.02	8.6	86.4	4.1	0.6	0.3
Experimental	8.87	1.01	7.36	0.47	0.03	0	11.4	83	5.3	0.3	0
Experimental	8.3	0.91	7.05	0.29	0.05	0	11	84.9	3.5	0.6	0
Experimental	6.64	0.76	5.53	0.28	0.06	0.01	11.4	83.3	4.2	0.9	0.2
Experimental	8.18	0.74	6.77	0.52	0.14	0.01	9	82.8	6.4	1.7	0.1
Mean	7.9	0.8	6.7	0.4	0.1	0.0	9.9	84.2	4.9	0.8	0.2
SD	0.7	0.1	0.5	0.1	0.0	0.0	1.2	1.4	1.2	0.4	0.1
SEM	0.3	0.0	0.2	0.0	0.0	0.0	0.4	0.5	0.4	0.2	0.1
